# Scrutinizes the Sustainable Role of Halophilic Microbial Strains on Oxygen-Evolving Complex, Specific Energy Fluxes, Energy Flow and Nitrogen Assimilation of Sunflower Cultivars in a Suboptimal Environment

**DOI:** 10.3389/fpls.2022.913825

**Published:** 2022-07-18

**Authors:** Fiza Ali, Xiangying Wei, Zamin Shaheed Siddiqui, Jianjun Chen, Hafiza Hamna Ansari, Danish Wajid, Zafar Iqbal Shams, Muhammad Waseem Abbasi, Urooj Zafar

**Affiliations:** ^1^Department of Botany, Stress Physiology Phenomics Centre, University of Karachi, Karachi, Pakistan; ^2^Institute of Oceanography, Minjiang University, Fuzhou, China; ^3^Environmental Horticulture Department and Mid-florida Research and Education Center, IFAS, University of Florida, Apopka, FL, United States; ^4^Institute of Environmental Studies, University of Karachi, Karachi, Pakistan; ^5^M.A.H. Qadri Biological Research Center, University of Karachi, Karachi, Pakistan; ^6^Department of Microbiology, University of Karachi, Karachi, Pakistan

**Keywords:** light harvesting, energy, bio-inoculation, halotolerant microbial strains, photochemical efficiencies

## Abstract

Environmental extremes such as hypersaline conditions are significant threats to agricultural productivity. The sustainable use of halophilic microbial strains was evaluated in plant in a salt stress environment. Oxygen-evolving complex (OEC), energy compartmentalization, harvesting efficiencies (LHE), specific energy fluxes (SEF), and nitrogen assimilation of oilseed crops (Sunflower cultivars) in a suboptimal environment was examined. Plants were grown in a plastic pot (15 ×18 cm^2^) containing sterilized (autoclaved at 120°C for 1 h) soil. Twenty-five ml suspension (10^7^ CFU/ml) each of *Bacillus cereus* strain *KUB-15* and *KUB-27* (accession number NR 074540.1) and *Bacillus licheniformis* strain *AAB9* (accession number MW362506), were applied *via* drenching method. Month-old plants were subjected to salt stress *via* gradual increment method. The energy compartmentalization of microbial inoculated plants exposed to salt stress revealed higher photosystem II (PSII) activity at the donor side, lesser photo-inhibition, and increased performance of oxygen-evolving complex compared to control. High potassium (K^+^) and low sodium (Na^+^) ions in treated leaves with the activated barricade of the antioxidant system stimulated by *Bacillus* strains favored enhanced photochemical efficiency, smooth electron transport, and lesser energy dissipation in the stressed plants. Moreover, the results reveal the increased activity of nitrite reductase (NiR) and nitrate reductase (NR) by microbial inoculation that elevated the nitrogen availability in the salt-stressed plant. The current research concludes that the application of bio-inoculants that reside in the hyper-saline environment offers substantial potential to enhance salt tolerance in sunflowers by modulating their water uptake, chlorophyll, nitrogen metabolism, and better photochemical yield.

## Highlights

- Halophilic microbes were isolated from the saline environment.
- Bacterial inoculation enhanced the light-harvesting efficiency and energy compartmentalization.
- Better osmotic regulation and reduced energy dissipation enhance salt tolerance.
- Cultivar Agsun−5264 was more physiologically tolerant than S-278.

## Introduction

Overpopulation, environmental extremes, and abiotic stressors have affected the agricultural soils around the globe (Hasanuzzaman et al., 2013). Environmental fluctuations affect the ecosystem in several ways, and hyper-saline landscapes threaten global food security by compromising agricultural productivity (Bharti et al., 2016). Salinity stress is considered a major threat that might limit productivity worldwide. It was noticed that exposure to soil salinity to plants initiates responses at both morph-physiological and molecular levels due to salt-induced ionic and osmotic stress. Understanding salt stress responses and tolerance mechanisms is vital for developing salt-tolerant crops and sustaining crop productivity in the future (Cho et al., 2014). Salt stress results in ionic imbalance, impaired cellular homeostasis, and hormonal imbalance in plants. This contributes to the excessive accumulation of reactive free radicals (ROS), and retarded plant growth leading to cell death (Mahajan and Tuteja, 2005). Yield loss of around 70% has been reported among various agro-economically vital crops across the globe, and one of them is the sunflower (Nolan et al., 2007; Hussain et al., 2019).

Sunflower (*Helianthus annuus*) is an oily seed crop and has significant importance in agricultural industries worldwide (Birck et al., 2017). Its cultivation is continuously expanding due to its premium oil and dietary fiber content (Khan et al., 2015). It is a moderate salt-tolerant crop and can adapt to salt stress to a certain extent, depending upon the species with their genetic modification. Nearly 522 cultivars of sunflowers are studied, and 30 are salt-resistant (Li et al., 2020). Salt stress is a greater concern in a low rainfall region as inadequate water does not leach out excessive salt from the root zone (Nolan et al., 2007). Therefore, biotechnologically favored approaches for sustainable agriculture to overcome the impact of climatic change to avert the menaces of saline stress. The formulation of bio-inoculant with the utilization of microbial resources is quite common to induce salt tolerance in crops (Mahanty et al., 2017).

A rhizobacterium exists in a saline soil and offers substantial potential to serve as a soil-friendly proxy for bio-fertilizers in a sub-optimal environment. These rhizobacteria are phyto-stimulants exhibit growth-promoting features on a wide range of hosts. They trigger nitrogen assimilation, potassium and phosphate solubilization, iron accessibility, and bioremediation to stimulate systemic resistance in the plant (Berg and Smalla, 2009; Drogue et al., 2012; Mahanty et al., 2017; Bhattacharyya et al., 2020).

A plethora of research spells out the importance of *Bacillus* as PGPMs in promoting plant growth and development against sub-optimal conditions (Chakraborty et al., 2013; Kasim et al., 2013; Radhakrishnan et al., 2017). However, PGPM-induced photochemical yield concerning photosynthetic performance and subsequent nitrogen metabolism in plants is yet to be explored. The rhizobacteria populated in brackish soil may have better tolerance and positively impact plants in water stress conditions. Consequently, it is assumed that the newly isolated bacterial strains mineralize the soil minerals into the bio-available form under stress conditions. It might cause an ultimate impact on light-harvesting efficiency and photosynthetic performance and thus boost nitrogen metabolism and physiological tolerance in plants. Light-harvesting efficiency, performance index, number of active reaction centers, and heat dissipation are the few attributes that are very important to demonstrate photosystem II functionality in an abiotic stress condition (Siddiqui et al., 2021). Similarly, the flow of electrons in the thylakoid membrane and the efficiency of the evolving oxygen complex were probed to understand the tolerance mechanisms aided by PGPM in a salt stress condition. Therefore, three novel strains, i.e., *Bacillus cereus* strain *KUB-15, B*. *cereus* strain *KUB-27*, and *B. licheniformis* strain *AAB9*, were isolated from the saline environment to scrutinize the sustainable effect of newly isolated bacterial strains on oxygen-evolving complex, energy harvesting compartmentalization, PS II efficiencies and nitrogen assimilation in two sunflower cultivars, i.e., Agsun-5264 and S-278 under salt stress conditions were studied.

## Materials and Methods

### Collection of Seeds

Seeds of sunflower (*Helianthus annuus* L.) in two cultivars, i.e., Agsun-5264 and S-278, were provided by the Department of Seed Certification, Government of Pakistan. The germplasm of both cultivars was sterilized for 10 min with a bleach solution (10% commercial bleach + 0.02% triton X-100) and then rinsed 3 times with distilled water. The experiment was performed under the greenhouse conditions (28–32/18–20°C of day and night temperature, respectively, and 60–70% of relative humidity).

### Bacterial Strain

Microbial isolates were collected from the University of Karachi. Two microbial isolates, i.e., *Bacillus cereus* strain *KUB-15* and *KUB-27* (accession number NR 074540.1), were obtained from the Department of Botany. *Bacillus licheniformis* strain *AAB9* (accession number MW362506) was collected from the Department of Microbiology, University of Karachi, Pakistan. *Bacillus* species were cultured and purified on a Nutrient Agar medium for 24 h at 37°C. The bacterial colonies were calculated by multiplying with the dilution factor (Yadav et al., 2010). The working stock was prepared by inoculating the *Bacillus* species into the sterile broth (25 ml), shaken vigorously, and raised at 37°C for the next 24 h. The bacterial suspension was adjusted to 10^7^ Colony Forming Unit (CFU) /mL per 1 kg soil.

### Soil Treatment With *Bacillus* Species (Drenching)

Ten Seeds of both cultivars were sown in a plastic pot (15 ×18 cm^2^) containing sterilized (autoclaved at 120°C for 1 h) soil. Twenty-five milliliter suspension (10^7^ CFU/ml) of respective *Bacillus* species were applied *via* the drenching method and distilled water (bacteria-free) for control. The optimal concentration of *Bacillus* was selected by considering the preliminary evaluation of plants. Thirty-day-old plants were subjected to salt stress *via* the gradual increment method (Umar et al., 2019). Two different concentrations of NaCl were selected for salt stress, i.e., 100 and 200 mM. The plants irrigated with distilled water serve as a control among stress treatments. Soil composition includes silt (16%), organic matter (0.14%), clay (10%), sand (73%), and nitrogen (0.008%). The soil had 7.9 pH and 1.71 dSm^−1^ of EC.

**Figure 1:**
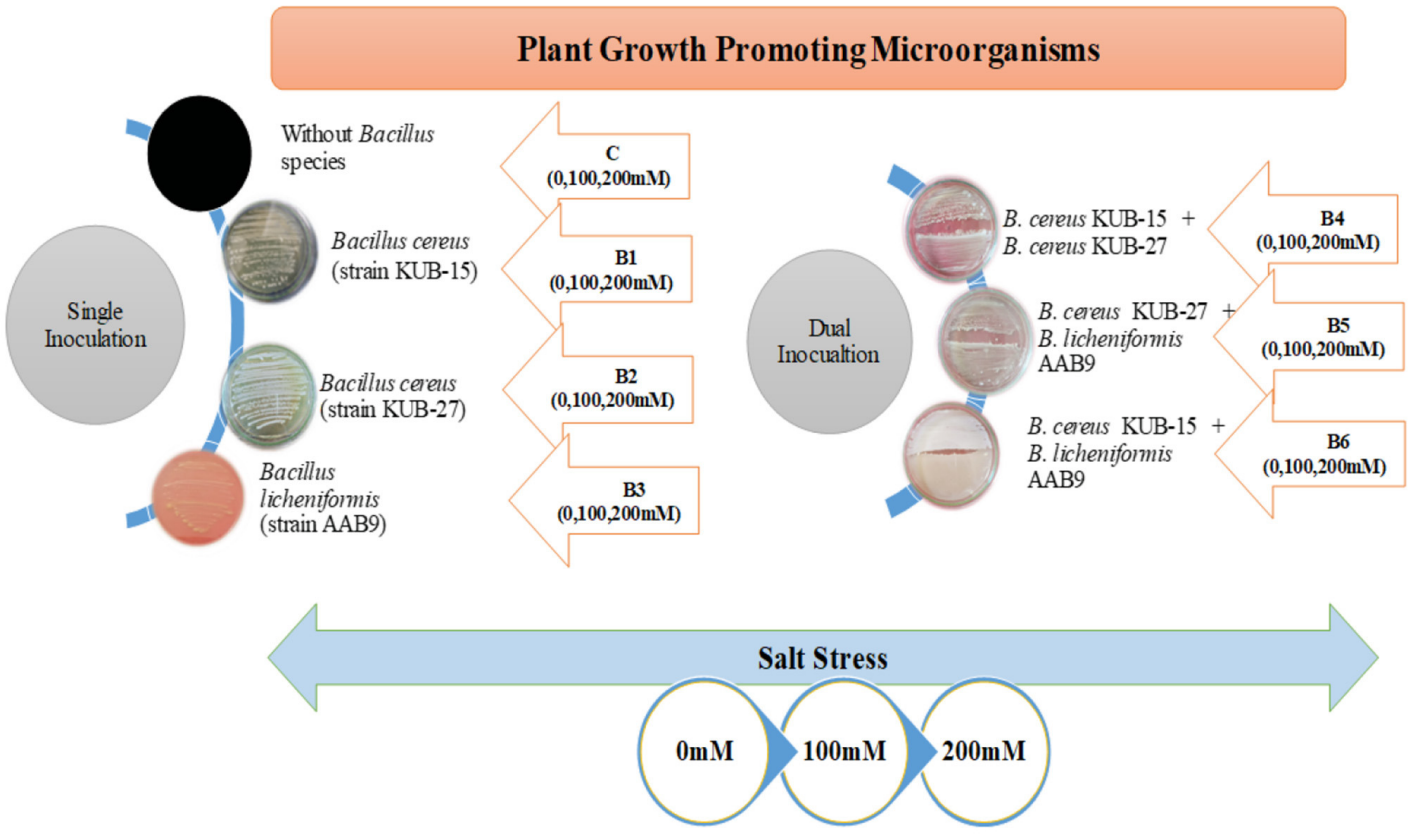
Schematic representation of bacterial treatments and stress application in plants.

All the treatments and the control were replicated four times and experienced a high light intensity of 28–35,000 lux, especially at noon. The greenhouse experiment includes the factorial design of seven treatments representing the application of bacterial isolates besides exposure to salt stress with their respective control (given in Figure 1). All the treatments and control were replicated four times and assessed for the growth and physiological aspects after harvesting.

### Morphological Characteristics

After harvesting the plants, their growth and biomass were measured and are given in Table 1. Ten plants from each treatment and control were randomly selected and washed with tap water to measure the seedling length and number of leaves. The length was measured with the help of a stainless scale. After measurement, the plant parts were chopped into small pieces and stored at low temperature for further analysis. Morphological illustrations of each treated plant and control were noted and demonstrated in Supplementary Figure 4A.

**Table 1:** Interactive effects of microbial strains on morphological traits of sunflower cultivars saline environment.

	**mM**	**SG (cm)**	**SFW (g)**	**SDW (g)**	**RFW (g)**	**RDW (g)**	**LFW (g)**	**LDW (g)**
**Agsun-5264**								
C	0	54.6 ± 3.66^a^	3.4 ± 0.15^a^	0.86 ± 0.01^a^	0.93 ± 0.01^a^	0.18 ± 0.02^a^	0.63 ± 0.04^a^	0.15 ± 0.01^a^
	100	36.6 ± 0.77^b^	2.3 ± 0.16^b^	0.43 ± 0.09^b^	0.43 ± 0.01^b^	0.05 ± 0.01^b^	0.36 ± 0.05^b^	0.09 ± 0.02^b^
	200	27.6 ± 4.55^c^	1.1 ± 0.17^c^	0.22 ± 0.02^c^	0.20 ± 0.02^c^	0.02 ± 0.01^c^	0.16 ± 0.04^c^	0.05 ± 0.01^c^
B1	0	62.3 ± 0.77^a^	4.5 ± 0.26^a^	1.03 ± 0.03^a^	1.53 ± 0.07^a^	0.70 ± 0.01^a^	0.80 ± 0.04^a^	0.53 ± 0.01^a^
	100	43.0 ± 1.13^b^	2.5 ± 0.14^b^	0.83 ± 0.01^b^	1.00 ± 0.03^b^	0.47 ± 0.05^b^	0.46 ± 0.07^b^	0.12 ± 0.01^b^
	200	39.0 ± 1.78^c^	1.8 ± 0.26^c^	0.63 ± 0.01^c^	0.70 ± 0.01^c^	0.15 ± 0.01^c^	0.20 ± 0.05^c^	0.09 ± 0.01^c^
B2	0	57.0 ± 0.33^a^	3.4 ± 0.21^a^	0.90 ± 0.01^a^	1.33 ± 0.04^a^	0.52 ± 0.01^a^	0.70 ± 0.05^a^	0.41 ± 0.01^a^
	100	41.3 ± 0.98^b^	2.4 ± 0.13^b^	0.60 ± 0.08^b^	0.80 ± 0.01^b^	0.18 ± 0.01^b^	0.56 ± 0.06^b^	0.10 ± 0.01^b^
	200	31.6 ± 1.87^c^	1.2 ± 0.12^c^	0.33 ± 0.08^c^	0.46 ± 0.04^c^	0.05 ± 0.01^c^	0.23 ± 0.09^c^	0.05 ± 0.01^c^
B3	0	67.0 ± 4.15^a^	5.1 ± 0.10^a^	1.36 ± 0.10^a^	1.73 ± 0.09^a^	0.70 ± 0.03^a^	1.13 ± 0.03^a^	0.63 ± 0.01^a^
	100	46.0 ± 2.75^b^	2.8 ± 0.11^b^	0.73 ± 0.16^b^	1.33 ± 0.08^b^	0.42 ± 0.01^b^	0.93 ± 0.03^b^	0.29 ± 0.01^b^
	200	39.3 ± 4.05^b^	2.0 ± 0.11^c^	0.62 ± 0.01^b^	0.96 ± 0.01^c^	0.15 ± 0.08^c^	0.73 ± 0.01^c^	0.14 ± 0.01^c^
B4	0	64.0 ± 3.35^a^	4.5 ± 0.11^a^	0.90 ± 0.01^a^	1.53 ± 0.08^a^	0.52 ± 0.03^a^	0.91 ± 0.01^a^	0.62 ± 0.01^a^
	100	44.6 ± 1.27^b^	2.4 ± 0.11^b^	0.73 ± 0.02^b^	0.90 ± 0.01^b^	0.18 ± 0.06^b^	0.56 ± 0.01^b^	0.29 ± 0.01^b^
	200	36.9 ± 0.39^c^	1.7 ± 0.11^c^	0.63 ± 0.01^c^	0.73 ± 0.01^c^	0.06 ± 0.01^c^	0.43 ± 0.01^c^	0.12 ± 0.01^c^
B5	0	54.3 ± 3.86^a^	4.0 ± 0.11^a^	0.75 ± 0.10^a^	1.60 ± 0.20^a^	0.57 ± 0.01^a^	0.90 ± 0.01^a^	0.60 ± 0.02^a^
	100	45.8 ± 2.46^b^	2.5 ± 0.12^b^	0.57 ± 0.09^b^	0.70 ± 0.04^b^	0.22 ± 0.05^b^	0.48 ± 0.02^b^	0.28 ± 0.01^b^
	200	34.6 ± 5.22^c^	1.6 ± 0.12^c^	0.41 ± 0.11^c^	0.56 ± 0.01^c^	0.06 ± 0.08^c^	0.33 ± 0.02^c^	0.12 ± 0.01^c^
B6	0	73.0 ± 3.15^a^	6.8 ± 0.11^a^	2.36 ± 0.11^a^	2.53 ± 0.11^a^	0.83 ± 0.07^a^	1.93 ± 0.09^a^	0.70 ± 0.07^a^
	100	56.0 ± 2.13^b^	3.9 ± 0.11^b^	1.61 ± 0.01^b^	1.60 ± 0.27^b^	0.45 ± 0.05^b^	1.26 ± 0.08^b^	0.38 ± 0.09^b^
	200	45.0 ± 2.65^c^	2.4 ± 0.11^c^	0.97 ± 0.01^c^	1.13 ± 0.33^b^	0.17 ± 0.06^c^	0.96 ± 0.06^c^	0.15 ± 0.01^c^
**S-278**								
C	0	39.9 ± 0.88^a^	2.8 ± 0.15^a^	0.60 ± 0.01^a^	0.63 ± 0.04^a^	0.15 ± 0.01^a^	0.56 ± 0.01^a^	0.12 ± 0.04^a^
	100	26.6 ± 1.32^b^	1.8 ± 0.18^b^	0.30 ± 0.03^b^	0.36 ± 0.07^b^	0.09 ± 0.01^b^	0.16 ± 0.01^b^	0.05 ± 0.01^b^
	200	21.6 ± 0.30^c^	1.0 ± 0.07^c^	0.15 ±0.03^c^	0.16 ± 0.08^c^	0.05 ± 0.01^c^	0.11 ± 0.01^c^	0.03 ± 0.01^b^
B1	0	52.3 ± 1.54^a^	3.3 ± 0.09^a^	0.86 ± 0.01^a^	0.80 ± 0.01^a^	0.53 ± 0.01^a^	0.74 ± 0.04^a^	0.27 ± 0.01^a^
	100	37.0 ± 1.50^b^	2.0 ± 0.17^b^	0.43 ± 0.01^b^	0.46 ± 0.01^b^	0.12 ± 0.01^b^	0.49 ± 0.03^b^	0.12 ± 0.04^b^
	200	28.0 ± 1.10^c^	1.5 ± 0.03^c^	0.20 ± 0.01^c^	0.20 ± 0.03^c^	0.09 ± 0.02^c^	0.33 ± 0.04^c^	0.06 ± 0.01^c^
B2	0	43.0 ± 1.02^a^	2.9 ± 0.09^a^	0.75 ± 0.06^a^	0.70 ± 0.03^a^	0.41 ± 0.03^a^	0.67 ± 0.03^a^	0.39 ± 0.01^a^
	100	33.0 ± 1.15^b^	1.9 ± 0.09^b^	0.40 ± 0.05^b^	0.56 ± 0.07^b^	0.10 ± 0.01^b^	0.42 ± 0.05^b^	0.10 ± 0.01^b^
	200	27.0 ± 1.34^c^	1.0 ± 0.09^c^	0.20 ± 0.05^c^	0.23 ± 0.09^c^	0.05 ± 0.01^c^	0.28 ± 0.03^c^	0.05 ± 0.01^c^
B3	0	57.2 ± 4.11^a^	3.5 ± 0.09^a^	0.70 ± 0.04^a^	1.13 ± 0.07^a^	0.63 ± 0.03^a^	0.98 ± 0.03^a^	0.46 ± 0.01^a^
	100	40.3 ± 3.11^b^	2.1 ± 0.09^b^	0.60 ± 0.01^b^	0.93 ± 0.06^b^	0.29 ± 0.01^b^	0.72 ± 0.03^b^	0.12 ± 0.01^b^
	200	30.0 ± 2.89^c^	1.4 ± 0.09^c^	0.40 ± 0.01^c^	0.73 ± 0.04^c^	0.14 ± 0.01^c^	0.48 ± 0.04^c^	0.05 ± 0.01^c^
B4	0	56.6 ± 3.11^a^	3.8 ± 0.01^a^	0.75 ± 0.09^a^	0.90 ± 0.01^a^	0.62 ± 0.01^a^	0.71 ± 0.01^a^	0.46 ± 0.01^a^
	100	36.3 ± 2.03^b^	2.0 ± 0.01^b^	0.40 ± 0.01^b^	0.56 ± 0.01^b^	0.29 ± 0.01^b^	0.49 ± 0.01^b^	0.12 ± 0.01^b^
	200	30.0 ± 1.33^c^	1.5 ± 0.09^c^	0.26 ± 0.05^c^	0.43 ± 0.01^c^	0.12 ± 0.01^c^	0.36 ± 0.02^c^	0.05 ± 0.01^c^
B5	0	51.6 ± 2.44^a^	3.1 ± 0.01^a^	0.86 ± 0.01^a^	0.90 ± 0.01^a^	0.60 ± 0.01^a^	0.67 ± 0.04^a^	0.27 ± 0.01^a^
	100	34.9 ± 3.34^b^	1.9 ± 0.01^b^	0.43 ± 0.01^b^	0.48 ± 0.01^b^	0.28 ± 0.04^b^	0.30 ± 0.01^b^	0.12 ± 0.01^b^
	200	28.3 ± 1.11^c^	1.2 ± 0.10^c^	0.20 ± 0.01^c^	0.33 ± 0.01^c^	0.12 ± 0.07^c^	0.31 ± 0.01^b^	0.06 ± 0.01^c^
B6	0	64.0 ± 1.96^a^	4.7 ± 0.10^a^	1.13 ± 0.01^a^	1.93 ± 0.05^a^	0.70 ± 0.07^a^	0.98 ± 0.07^a^	0.70 ± 0.01^a^
	100	45.9 ± 2.12^b^	2.4 ± 0.12^b^	0.90 ± 0.01^b^	1.26 ± 0.01^b^	0.38 ± 0.07^b^	0.72 ± 0.04^b^	0.18 ± 0.01^b^
	200	37.0 ± 2.23^c^	1.7 ± 0.15^c^	0.73 ± 0.01^c^	0.96 ± 0.01^c^	0.15 ± 0.01^c^	0.48 ± 0.04^c^	0.08 ± 0.01^b^

*Data presented are the mean ± standard error. Symbol C = Plants without Bacillus species, B1 = Plants with Bacillus cereus strain KUB-15, B2 = Plants with Bacillus cereus strain KUB-27, B3 = Plants with Bacillus licheniformis strain AAB9, B4 = Plants with B. cereus KUB-15+ B. cereus 27, B5 = Plants with B. cereus KUB-27+ B. licheniformis AAB9, B6 = Plants with B. cereus KUB-15+ B. licheniformis AAB9. 0, 100, and 200 shows the salinity concentration in mM*.

*The superscript defines the significant differences among the treatments*.

### Stomatal Conductance (SC) and Chlorophyll Content Index (CCI)

Ten intact leaves were randomly selected from each treatment and control to measure the stomatal conductance (SC), and chlorophyll content index (CCI). For CCI, portable and non-destructive device (CCM-200; Opti-Sciences Inc., Hudson, NH, USA) was used. Fully expanded leaves were taken for chlorophyll content measurements.

Decagon leaf Porometer (Model SC-1) was used to measure the stomatal conductance. The leaves were placed in between the sensor head of the portable device. The stomatal conductance (mmol m^−2^ s^−1^) and temperature were displayed on the device screen.

### Fluorescence Observation

Sixteen fully extended leaves of each treatment and control were selected to observe fluorescence traits using an Opti-science Fluorometer (Model OS30p^+^; Hudson, USA). The leaves were dark-adapted for 30 min by attaching the leaf clips to the leaves. The actinic light source (660 nm) was used to illuminate the leaves, which have an intensity of 3,500 μmol m^−2^ s^−1^ for 1 s. The data were then transferred to the computer by a connecting cord. Fluorescence parameters were assessed and modified according to the protocols of Stirbet et al. (2018), Strasser et al. (2010), and Maxwell and Johnson (2000).

### Osmotic Potential (O.P) and Relative Water Content (RWC)

Twenty Leaves from each control and treatment were randomly selected and frozen in liquid nitrogen to estimate the osmotic potential of the leaves. The grounded leaves (cell sap) were then centrifuged at 14,000 rpm for 15 min at 4°C. Osmotic potential of expressing cell sap of the tissues was noted by Osmometer type 6 Loser Messtechnik, Berlin, Germany. The samples' osmotic potential values were then converted into the unit bar (Turner, 2018). For relative water content measurement, fresh leaf samples were used. Five hundred mg of fresh weight was used. The samples were then kept in distilled water for 24 h to obtain the turgid weight (T.W) and then dried at 72°C for 48 h to obtain dry weight (D.W) to calculate relative water content (Barrs and Weatherley, 1962).

### Electrolyte Leakage

0.5 g of leaf blades were cut into the small disc by using a cork borer and contained in a bolted tube having double distilled water (20 ml) for the determination of the electrolyte leakage (%) (Lutts et al., 1996). The sample was incubated at 25°C for 24 h, and then a conductivity meter (CDM), mentioned as EC_1_, recorded the sample's electrical conductivity. The sample was boiled at 95°C for 30 min to note the solution's electrical conductivity as EC_2_. The following equation was used to determine the Electrolyte Leakage (%) as the ratio of EC_1_ and EC_2_:


$$\begin{array}{l} EC\;Leakage\;\left(\%\right)=\left(\frac{EC_{2}-EC_{1}}{EC_{1}}\right)\times 100 \end{array}$$

### H_2_O_2_ Production and Thio-Barbituric Acid Reactive Substances (TBARS Content)

Production of hydrogen peroxide (H_2_O_2_) was estimated in leaf samples of each treatment and control plant (Velikova et al., 2000). One hundred milligram of fresh leaf samples were homogenized with 0.1% trichloroacetic acid (3%) in an ice bath and then centrifuged for 15 min at 12,000 g. Later in 0.5 ml of supernatant, 0.5 ml of phosphate buffer (10 mM) with pH 7.0, and 1 ml of potassium iodide (1 M) were added. The absorbance was recorded at 390 nm and the content of H_2_O_2_ was calculated using a standard curve expressed in μmol g^−1^ FW (Dhindsa et al., 1981). Protocols were used to estimate TBARS content. The frozen sample (100 g) was homogenized with trichloroacetic acid (TCA 0.5%). The supernatant was then subjected to centrifugation for 15 min at 12,600 g (rev/min). Three thousand microliters of reagent solution which includes TCA (20%) and Thio-barbituric acid (0.5%) were added to a clear supernatant (1,000 μl) and then kept for 30 min in a water bath (100°C) and cooled at RT. Thiobarbituric acid reactive substances (TBARS)-TBA extinction coefficient was recorded at 532 nm as 155 mmol/cm. The TBARS content was calculated using the formula:


$$\begin{array}{l} TBARS\;\left(\text{μ}M\right)\;Concentration\;\\ =\;\frac{\left(Absorbance_{532}-\;Absorbance_{600}\right)}{155} \end{array}$$

### Assay of Antioxidant Enzymes

Leaf samples (500 mg) were homogenized in liquid nitrogen. Later antioxidant enzymes were extracted with 12 mL of extraction buffer (Tris-HCl pH 6.8, 10 mL DDT, 0.1 mM EDTA, 50 mg PVP). The mixture was then centrifuged at 4C for 15 min at 3,354 g for total protein estimation (Bradford, 1976). The antioxidant enzymes, i.e., catalase (EC # 1.11.1.6) and Superoxide Dismutase (EC # 1.15.1.1), were measured by the method of Patterson et al. (1984) and Beyer and Fridovich (1987), respectively.

### Nitrate Reductase Activity and Nitrite, Nitrate Estimation

Small pieces of leaves (200 mg) were cut and incubated with 5 mL of 0.1 M Phosphate buffer solution consisting of propanol (0.1%) and KNO_3_ (0.1 M), pH 7.2 under anaerobic and dark conditions. For assessing nitrate content in assay solution, 2,000 μL of color development reagent containing 1,000 μL each of 0.02% *N-*(1-naphthyl) ethylene diamine hydrochloride (NDD) and 1% sulfanilamide in HCL were added to 100 μL of the assay solution. The O.D of the samples was recorded at 540 nm (μmole NO_2_ h^−1^ g^−1^ fresh weight expressed the enzyme's activity). Nitrate reductase (NR) and nitrite (NIR) activity was measured as suggested by Srivastava (1975) and Losada and Paneque (1971) based on the nitrate reduction at 540 nm. Total nitrite and nitrate-N content were estimated by acid digestion (Nerdy and Putra, 2018).

### Ion Analysis

A flame photometer (Jenway PFP7, UK) was used for ion analysis (Na^+^ and K^+^ ions). Ten milliliter of 2 N HCl (diluted with deionized water) was used to digest the oven-dried leaf samples, and which were then analyzed through a flame photometer suggested by Chapman and Pratt (1961)).

### Statistical Analysis

All treatments and control were subjected to SPSS version 20 for the statistical analysis of the current data. The Bonferroni *post-hoc* test was applied to differentiate significant differences among the mean values of different treatments and presented as small alphabets on the bar graphs (*p* ≤ 0.05).

## Results

### Effect of Microbial Strain on Growth and Biomass Accumulation

Growth parameters and seedling biomass were retarded under salt stress. The plants were severely affected under 150 mM compared to 75 mM salt stress. It was observed that the inoculation of halophilic bacteria in salt-treated cultivars revealed a significant increase in plant growth and biomass accumulation. Table 1 showed that *Bacillus licheniformis* in single inoculation (B3) and in combination (B6 and B5) had more pronounced effects in sunflower cultivars than *Bacillus cereus* strains (B1, B2, and B4) under a salt-stress environment. The growth and biomass accumulation of cultivar Agsun-5264 was better than in S-278 under salt stress conditions. Leaf dry and fresh weight was greatly improved in both cultivars under salt stress conditions.

### Physiological Response

**Figure 2:**
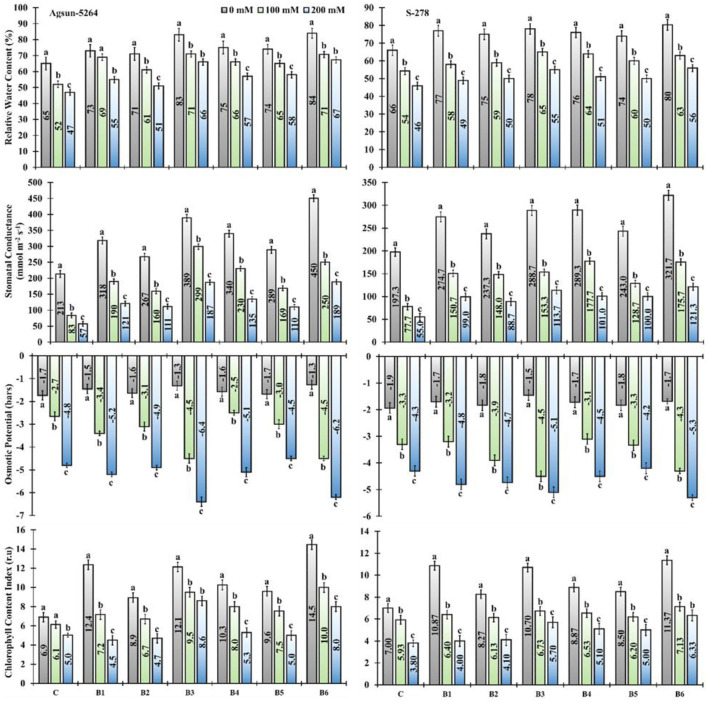
Effect of halophilic microbial strains on the relative water content (RWC), stomatal conductance (S.C), osmotic potential (O.P), and chlorophyll content index (CCI) of sunflower cultivars under salinity stress. Vertical line on the bar represents mean standard error (S.E). Similar alphabet shows non-significant difference at *p* ≤ 0.05 (*Bonferroni post-hoc test*) between treatments. Symbols on the horizontal axis represents plants: C = Without *Bacillus* species, B1 = *Bacillus cereus* strain *KUB-15*, B2 = *Bacillus cereus* strain *KUB-27*, B3 = *Bacillus licheniformis* strain *AAB9*, B4 = *KUB-15* + *KUB-27*, B5 = *KUB-27* + *AAB9* and B6 = *KUB-15* + *AAB9*. Legends demonstrate that 0 mM = Control plants without salinity stress, 100 mM = Plants treated with 100 mM NaCl, and 200 mM = Plants treated with 200 mM NaCl.

The application of newly isolated bacterial strains substantially improved relative water content (RWC), stomatal conductance (SC), and chlorophyll content index (CCI) of both sunflower cultivars exposed to salt stress (Figure 2). Application of single/dual inoculation of bacterial strains showed a substantial increase in the physiological parameters of both sunflower cultivars compared to uninoculated cultivars in a saline environment. It was evident from the data that the application of *B. licheniformis* (B3) improved 36–40% in RWC, 258–228% in SC, and 54–109% in CCI of Agsun-5264 under salt stress conditions. While 35–43% RWC, 200–231% SC, and 63–95% CCI increase were observed in the combined treated sample (B6) (Supplementary Table 2). Similarly, the inoculation of B3 and B6 pointedly decreased the osmotic potential in cultivar Agsun-5264 compared to other treatments under salinity stress conditions (Figure 2).

### Photochemical Efficiencies

**Figure 3:**
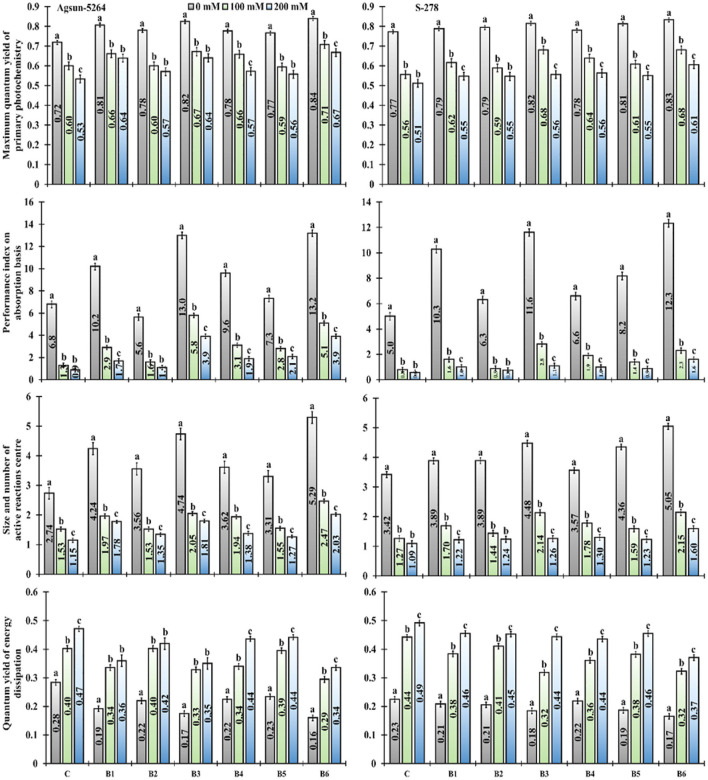
Effect of halophilic microbial strains on Maximum quantum yield of primary photochemistry (F_V_/F_M_), Performance index on absorption basis (PI_ABS_), Size and number of active reaction center (F_V_/F_O_) and Quantum yield of energy dissipation (F_O_/F_M_) of sunflower cultivars during salinity stress. Vertical line on the bar represents mean standard error (S.E). Similar alphabet shows non-significant difference at *p* ≤ 0.05 (*Bonferroni post-hoc test*) between treatments.

It was noted that the performance index (PI_ABS_), dark-adapted quantum yield (F_V_/F_M_ ratio), the number of the active reaction center of photosynthetic apparatus (F_V_/F_O_) of salt-treated cultivars were greatly reduced (Figure 3). The salt stress did not only decrease the cultivars' performance index but also increased energy dissipation (F_O_/F_M_). It was evident from the data the magnitudes of the negative impact of salinity on photochemical traits like F_V_/F_M_ ratio, PI_ABS_, and F_V_/F_O_ ratio was mitigated due to bacterial application, application of *B. licheniformis* (B3) in salt stress, *which* showed 11–20% in F_V_/F_M_, 346–343% in PI_ABS_, and 34%, to 57% increase in Fv/F_O_. In comparison, 17–25% F_V_/F_M_, 292–343% PI_ABS_, and 61–76% Fv/F_O_ were increased in combined treatment (B6), compared to un-inoculated control plants (Supplementary Table 2). The single (B3) and dual (B6) application of newly isolated bacterial stain *B. licheniformis* made a more positive impact on Agsun-5264, showed substantial improvement in the F_V_/F_M_ ratio, PI_ABS_, F_V_/F_O_ ratio, and declined in F_O_/F_M_ under salt stress conditions (Figure 3).

**Figure 4:**
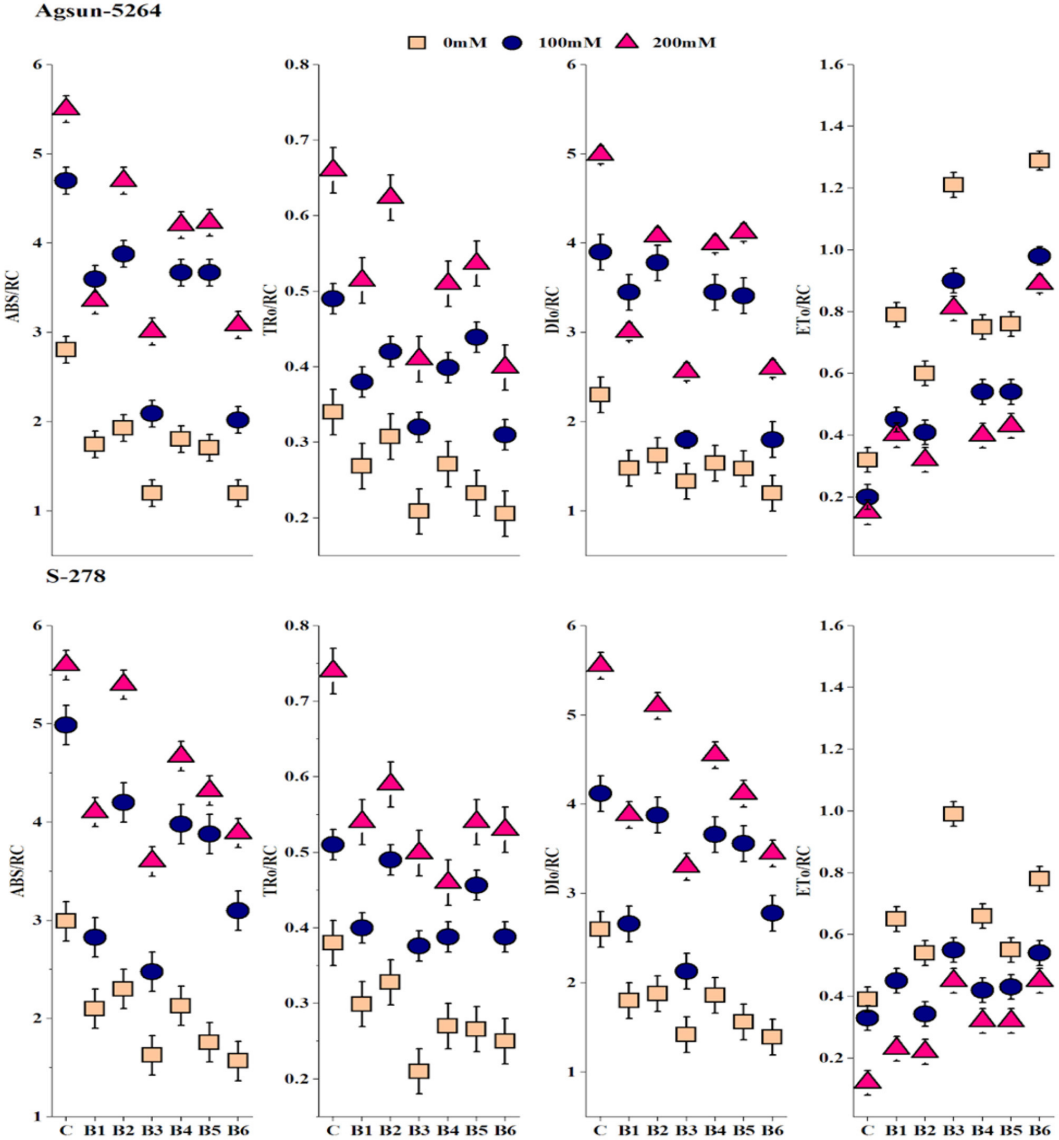
Effect of halophilic microbial strains on changes in specific energy fluxes per reaction center including Absorption per reaction center (ABS/RC), Trapping per reaction center (TR_O_/RC), Electron transport per reaction center (ET_O_/RC) and Dissipation per reaction center (DI_O_/RC) of sunflower cultivars during salinity stress. Vertical line on the bar represents mean standard error (S.E). Similar alphabet shows non-significant difference at *p* ≤ 0.05 (*Bonferroni post-hoc test*) between treatments.

Both treated cultivars' specific and phenomenological energy fluxes were calculated on reaction center (RC) and cross-section (CS) basis. It was evident from the data that electron transport per active reaction center (ET_O_/RC or CS), absorbance per reaction center (ABS/RC or CS), maximum trapped exciton flux per active PSII (TR_O_/RC or CS), and dissipated energy flux per active reaction center (DI_O_/RC or CS) of sunflower cultivars were greatly affected in saline condition, demonstrated significant differences among cultivars (Figure 4 and Supplementary Figure 1). Application of bacterial strains B3, and its combination B6 caused substantial improvement in ABS/CS, TR_O_/CS, and ET_O_/RC or CS under salt stress conditions. However, the magnitude of energy loss or energy dissipation was declined due to bacterial application B3 and B6 in particular. The bacterial application did a more pronounced effect on Agsun-5264 under a salt-stress environment.

Some fluorescence parameters like V_I_, 1-V_I_, V_J_,1-V_J_, and W_k_ were assessed in salinity stress. The value of relative variable Chl a fluorescence at I-step (V_I_), relative variable Chl a fluorescence at J-step (V_J_), and ratio of variable fluorescence at K-step to the amplitude F_J_-F_O_ (W_k_) were increased. At the same time, 1-V_I_ and 1-V_J_ decreased in both the cultivars compared to the control (Supplementary Figure 2). But in comparison, among the single/dual inoculation of PGPMs, B3 and B6 are substantially lower down the V_I_, V_J_, and W_k_ in Agsun-5264 compared to cultivar S-278 under a saline environment (Supplementary Figure 2). Meanwhile, PGPMs significantly increase the 1-V_I_ and 1-V_J_ in Agsun-5264 than S-278 under stress.

Energy pipeline model showing the proportion of specific energy fluxes per active photosystem II. The model was developed to compare the efficacy of bacterial strain regarding the ABS/RC, ET_O_/RC, TR_O_/RC, DI_O_/RC, and NiR activity in the salt-stressed cultivar with or without PGPMs. In comparison, among the treatments of PGPMs, B3 and B6 greatly improved ET_O_/CS and NiR in cultivar Agsun-5264 than S-278. The membrane model also showed the application of B3 and B6 substantially maintained the ABS/RC, TR_O_/RC, and DI_O_/RC in cultivar Agsun-5264 than S-278.

**Figure 5:**
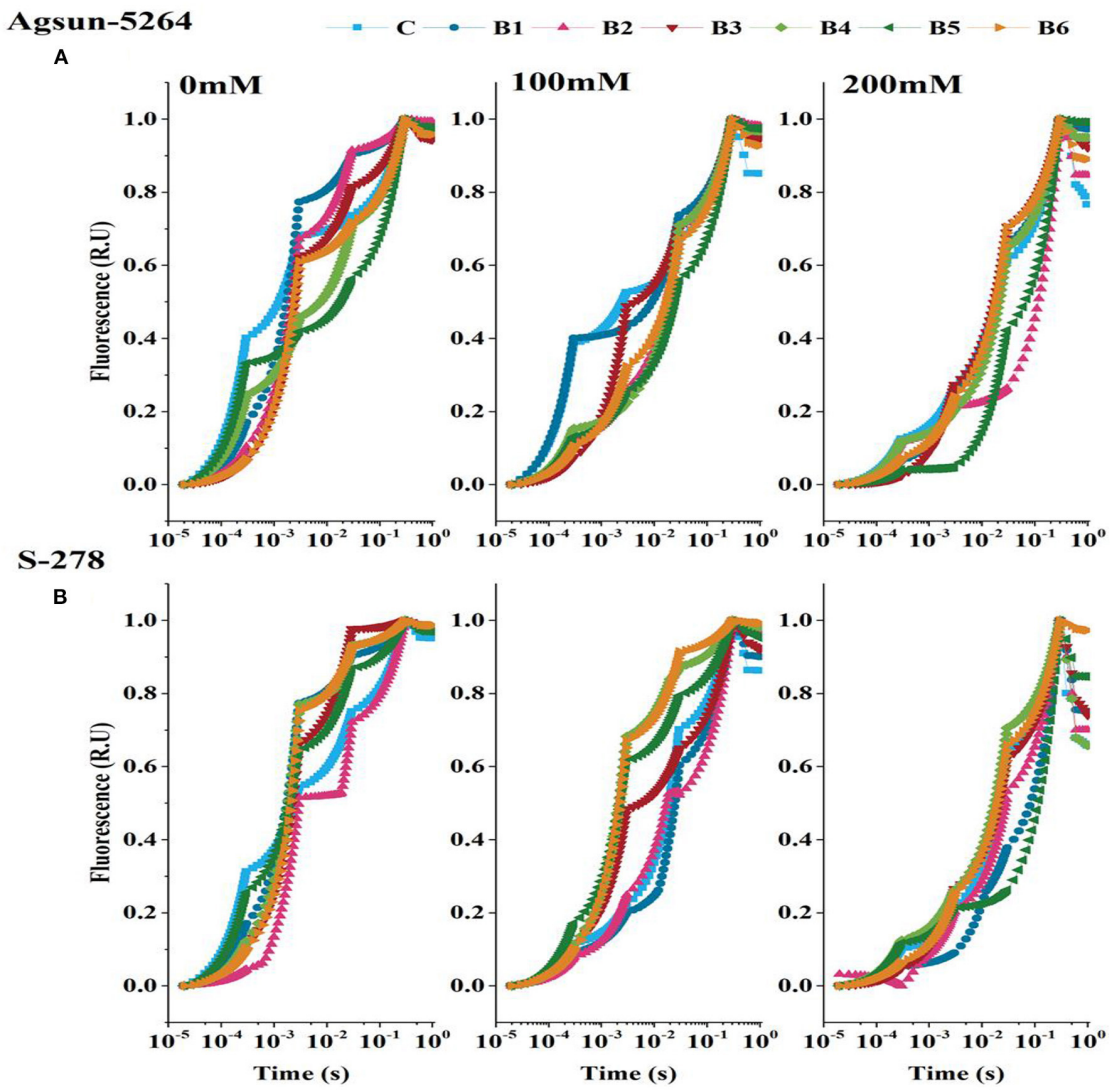
Effect of halophilic microbial strains on Chlorophyll a fluorescence induction curve (OJIP) of sunflower cultivars. **(A)** Agsun-5264 and **(B)** S-278 under salinity stress.

OJIP-transient or induction curve was stable with PGPMs in both cultivars under a stress environment (Figure 5). However, compared to the single/dual inoculation of PGPMs, B3 and B6 highly retained OJIP-transient or induction curve in Agsun-5264, indicating an effective enhancement in photosynthetic rate S-278 under stress.

### ROS Scavenging and Antioxidant System

In particular, usage of single/dual inoculation of PGPMs, B3, and B6 substantially lower the magnitude of MDA (53 and 51%) and H_2_O_2_ (43 and 42%) content in Agsun-5264 under salt stress condition, indicating lesser membrane damage compared to other treatments. Similar trend was observed among the cultivar S-278 (Supplementary Table 2). The application of PGPMs revealed that enzymes catalase (CAT) and superoxide dismutase (SOD) activities was elevated in both sunflower cultivars with the increase in NaCl concentration. However, Agsun-5264 showed maximum SOD and CAT activity (72–74% and 101–110%) than S-278 due to B3 and B6 inoculation. It has appeared that the presence of PGPMs in saline environments increased the antioxidant enzyme activity compared to saline medium without PGPMs.

### Nitrogen Metabolism and Related Key Enzymes

**Figure 6:**
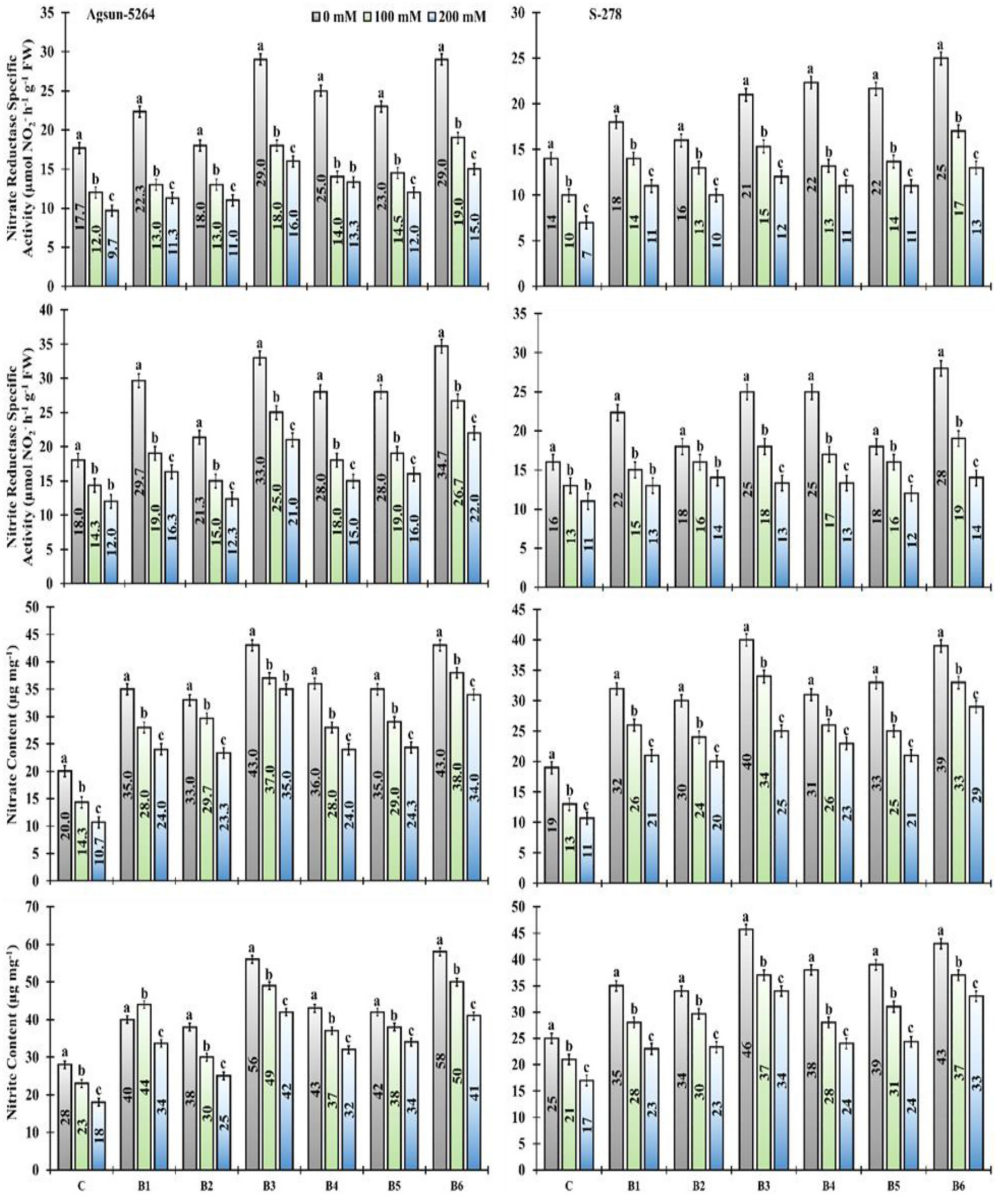
Effect of halophilic microbial strains on changes in Nitrate reductase specific activity (NR), Nitrite reductase specific activity (NiR), Nitrate content and Nitrite content of sunflower cultivars under salinity stress. Vertical line on the bar represents mean standard error (S.E). Similar alphabet shows non-significant difference at *p* ≤ 0.05 (*Bonferroni post-hoc test*) between treatments.

The nitrite (NiR) and nitrate (NR) reductase activity significantly decreased under salt stress. It was noted that the magnitude of nitrite and nitrate content of sunflower cultivars decreased due to varying degrees of salt stress. However, PGPMs, B3, and B6 greatly increased the nitrite (NIR) and nitrate (NR) reductase activity under a salt stress environment. The nitrite and nitrate content in Agsun-5264 was increased compared to S-278 after PGPM inoculation. The significant enhancement in nitrogen assimilation was observed when salt stress plant were subjected to B3 and B6 bio-inoculant treatments (Figure 6).

### Ionic Regulation

**Figure 7:**
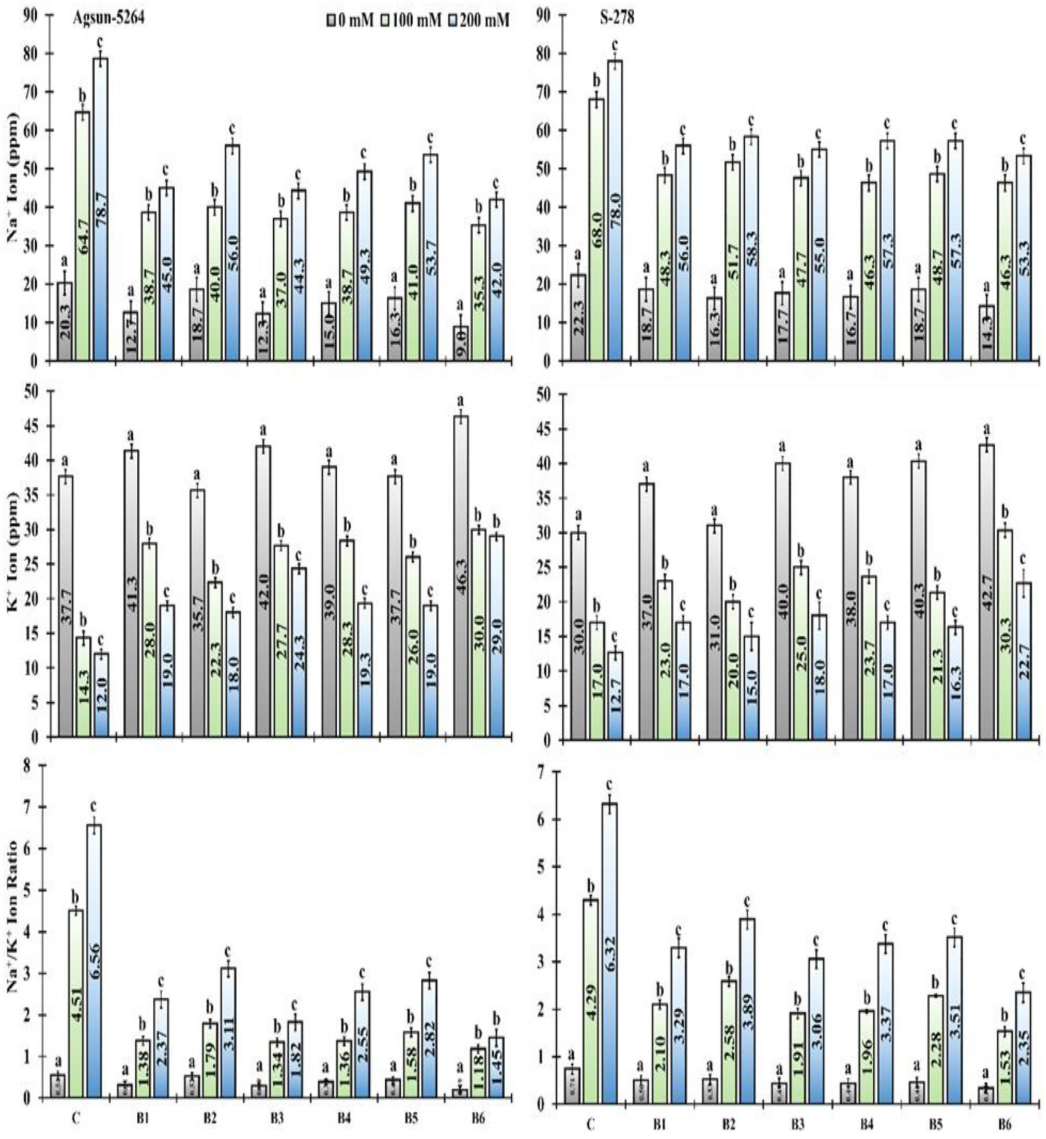
Effect of halophilic microbial strains on changes in sodium ion (Na^+^), potassium ion (K^+^), and Na^+^/K^+^ ratio of sunflower cultivars under salinity stress. Vertical bar lines express standard error (S.E) and similar letters on the error bar represent non-significant difference at *p* ≤ 0.05 (*Bonferroni post-hoc test*) between treatments.

PGPMs decreased the sodium (Na+) ion concentration and Na^+^/K^+^ ratio in both sunflower cultivars under salinity stress (Figure 7). However, in comparison among the single/dual inoculation of PGPMs, B3 and B6, in particular, greatly reduced the Na^+^ ion and Na^+^/K^+^ ratio in Agsun-5264 compared to other cultivar S-278 under saline environment (Supplementary Table 2). PGPMs significantly increase the K^+^ accumulation in both cultivars under stress environments (Figure 7). It was noticed that the utilization of PGPMs, like, B3 and B6, substantially improved the K^+^ ratio in Agsun-5264 compared to S-278 under stress conditions.

## Discussion

The exploitation of halotolerant bio-inoculants such as PGPMs to deal with stress has emerged to improve the productivity of crops (Grover et al., 2011). The present study investigates the potential use of PGPMs in hypersaline conditions to induce salt tolerance in crop plants. Salt tolerance was assessed using photochemical features and subsequent nitrogen metabolism.

Sunflower cultivars showed significant variation in growth characteristics under stress condition (Table 1). Apparently application of newly isolated bacterial strains *B. licheniformis* (B3) or in dual condition (B6) lessens the negative impact of salt stress in sunflower cultivars. The results revealed that single/dual inoculation of *B. licheniformis* enhanced the growth and biomass in Agsun-5264. It was supported by the literature that the dual inoculants such as *Azospirillum brasilense* and *Pseudomonas fluorescens* enhanced height, biomass, yield, and antioxidant enzyme activity in potato plants under abiotic stress conditions (Trdan et al., 2019). Moreover, single or dual PGPMs inoculation increased RWC in plants indicating the microbial association with plant that can weaken abiotic stress conditions (Igiehon and Babalola, 2021). The investigation showed that Agsun-5264 had greater stomatal conductance with the use of *B. licheniformis* under salinity stress, proving a fast-growth strategy to deal with stress. Sustaining a significant quantity of relative water content and stomatal conductance in leaves is a vital strategy for tackling abiotic stress, and stress-tolerant species also maintain adequate relative water content in a stress environment (Chartzoulakis et al., 2002; Cho et al., 2014; Siddiqui et al., 2014; Yao et al., 2018). Similarly higher chlorophyll content in Agsun-5264 due to *B. licheniformis* application under saline conditions is likely to lead to robust photosynthesis. The higher photosynthesis in cultivar Agsun-5264 due to *B. licheniformis* indicates that salinity did not significantly damage the photosynthetic apparatus (Gong et al., 2014). The osmotic potential is enhanced in non-inoculated plants compared to the inoculated ones (Figure 2). The results endorse the findings of Siddiqui et al. (2014), suggesting higher Na+ ions accumulation due to higher osmotic potential that causes osmotic stress in crops. High osmotic potential reduces the plants' ability to absorb water from the soil (Machado and Serralheiro, 2017). The study showed lower osmotic potentials of Agsun-5264 due to the application of PGPMs B3 and B6, indicating retention of water from the soil under salinity stress conditions. Moreover, there might be the possibility of bacterial symbiosis with Agsun-5264 providing tolerance to the salt-treated plants that result in an alignment of the osmotic potential for cellular water retention and turgor maintenance, thus reducing the detrimental effects of salt stress by stabilizing the solute potential (Bai et al., 2019).

The cultivar Agsun-5264 divulged the higher FV/FM ratio value, PIABS, and FV/FO, representing the lower disruption in antenna complex and RuBP regeneration capability due to single/dual inoculations of B3 and B6 under salinity stress. It was evident from the data that the electron transport rate from Q_A_ to Q_B_ remained unaffected in sunflower cultivar under salt stress due to bio-inoculant. Furthermore, vibrant reaction centers, larger size, and the quantity of photosynthetic apparatus leads to lesser photodamage in saline environment (Umar et al., 2019). It further indicates that the application of *B. licheniformis* (B3) in salt stress conditions eliminates all reservations at the oxidation sides of PSI and PSII (Zaghdoudi et al., 2011). The present study reveals that *B. licheniformis* improves CCI value, which finally raised the PI_ABS_ under salinity stress conditions. Increased PI_ABS_ improved photochemical efficiency or smoothed electron transport chain, revealing that the system structure, photoinhibition of photosynthesis, potential activity, and function of PSII were not affected during severe stress conditions (Appenroth et al., 2003; Oukarroum et al., 2009; Kalaji et al., 2018). Similarly, *B. licheniformis* treated cultivar Agsun-5264 divulged low values of F_O_/F_M_, signifying a lower disruption in electron transport at the PSII donor side, and the plastoquinone reduction (Q_A_) rate was lower than the oxidation rate (Q_B_) (De Lucena et al., 2012; Umar et al., 2019).

Specific energy fluxes per active reaction center (RC) including ABS/RC, ET_O_/RC, TR_O_/RC, and DI_O_/RC divulged absorption, trapping, electron transfer to Q_A−_ to PQ, and energy flux dissipated into heat (Figure 4). A rapid decrease in DI_O_/RC and DI_O_/CS implied the bulk of energies was not dissipated in a heat form (Meng et al., 2016). *B. licheniformis* decreased the photoinhibition in Agsun-5264 associated with the lesser DI_O_/RC related to the higher F_V_/F_M_ ratio during salinity stress. Further, *B. licheniformis* treated Agsun-5264 reflected better ET_O_/RC. The absorption rate is higher than the energy utilization rate, suggesting that PSII may be quenched non-photochemically to prevent photodamage (Siddiqui et al., 2021). Meanwhile, phenomenological energy fluxes per CS, including ABS/CS, ET_O_/CS, and TR_O_/CS increased in cultivar Agsun-5264 due to utilization of B3 and B6, indicating that increases in the density of vibrant reaction centers finally improve the trapping efficiency of PSII (Siddiqui et al., 2021).

It is evident (Supplementary Figure 2) that Agsun-5264 treated with *B. licheniformis* enhances photochemical performance and has better tolerance against salt stress. Application of bio-inoculant B3 and B6 increased 1-V_I_ and 1-V_J_ and decreased V_I_ and V_J_ in Agsun-5264 under stress conditions. These results showed a smooth electrons flow from Q_A_ to Q_B_ and reduced assemblage of Q_A_ in PSII reaction centers (Da Browski et al., 2016). Further, B3 and B6 decreased 'K' step (W_K_) demonstrated the lowest harm in the oxygen-evolving complex (OEC), which led to a rise in the electron donation ability of the PSII donor side in cultivar Agsun-5264. The Wk values are associated with the temperature of the stress plants (Wen et al., 2005; Van Heerden et al., 2007).

**Figure 8:**
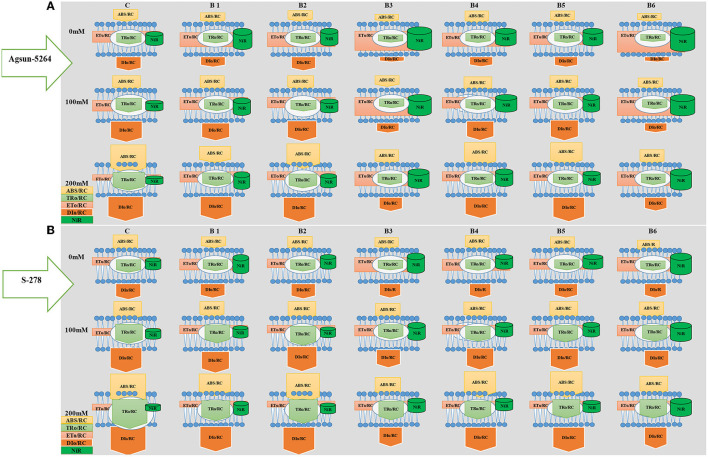
Energy pipeline model showing the proportion of specific energy fluxes per active PSII and NiR activity in the membrane of salt-stressed sunflower cultivars Agsun-5264 with or without microbial strains. In the membrane, ABS/RC, TR_O_/RC, ET_O_/RC, and DI_O_/RC indicate absorption, maximum trapped exciton flux per active PSII, electron transport, and dissipation flux, respectively. The value of each parameter can be seen in relative changes in the width of each arrow (*see* the color legend). The diagram exhibits the variation of ABS/RC, TR_O_/RC, ET_O_/RC, and DI_O_/RC, for seven treatments, namely, C = Without *Bacillus* species, B1 = *Bacillus cereus* strain *KUB-15*, B2 = *Bacillus cereus* strain *KUB-27*, B3 = *Bacillus licheniformis* strain *AAB9*, B4 = *KUB-15* + *KUB-27*, B5 = *KUB-27* + *AAB9*, and B6 = *KUB-15* + *AAB9*. The model was developed by the values of four fluxes and NIR activity. The values of each of the four fluxes and NIR activity can be compared between cultivars and among treatments. The model displays fluxes and NIR activity in different shapes; the size of each shape is proportional to the corresponding normalized value. **(A)** Agsun-5264 and **(B)** S-278.

Model (Figure 8) showed the tolerance level of the sunflower cultivar Agsun-5264 due to *B. licheniformis* by regulating the activities of PSII reaction centers and enhancing the chloroplasts' ability to absorb and dissipate energy is a key to increasing the salt tolerance. In this model, *B. licheniformis* increase of ET_O_/RC and NiR in cultivar Agsun-5264 indicates that the function and organization of PSII were less damaged and may be a defensive strategy to cope with salt stress. In contrast, *B. licheniformis* reduced ABS/RC and TR_O_/RC during salt stress conditions implying that the effective average absorption of antenna and trapping per active RC are lower due to some RC activation. *B. lichaniformis* decreased the photoinhibition in Agsun-5264 linked to lesser DI_O_/RC related to higher TRo/RC during salinity stress and increased regulation of PSII activities. The results demonstrate that average absorption and trapping per active RC rise due to the deactivation of a few RCs, and the total dissipation to the number of active RCs rises due to the high dissipation of dormant RCs. *B. licheniformis* enhanced nitrogen assimilation, improving chlorophyll formation and raising photosynthetic activity in cultivars Agsun-5264 during stress. The data regarding better OJIP transient curve and accumulation of active reaction centers of PSII in Agsun-5264 than S-278 (Figure 5), further strengthening the idea concerning adequate photosynthesis performance under stress conditions (Strasser et al., 2004; Umar et al., 2019).

**Figure 9:**
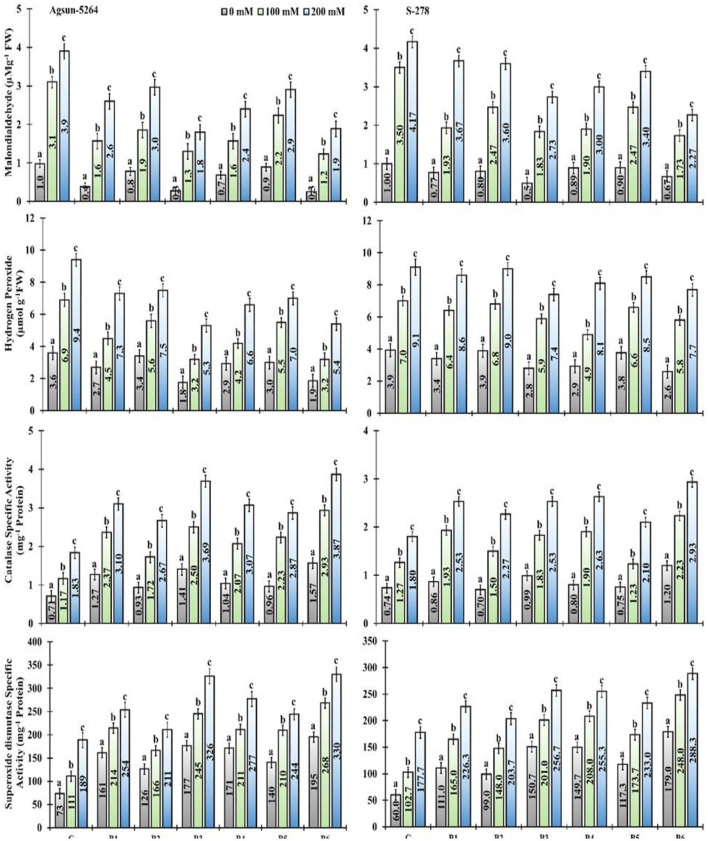
Effect of halophilic microbial strains on changes in Malondialdehyde (MDA), Hydrogen peroxide (H_2_O_2_), Catalase specific activity (CAT), and Superoxide dismutase specific activity (SOD) of sunflower cultivars under salinity stress. Vertical line on the bar represents mean standard error (S.E). Similar alphabet shows non-significant difference at *p* ≤ 0.05 *(Bonferroni post-hoc test)* between treatments.

In the present study, inoculated plants exhibited lower values of MDA, H_2_O_2_ content, and electrolyte leakage (E.L) under salt-stress conditions. Their values were distinctly higher in the non-inoculated plants (Figure 9 and Supplementary Figure 3). The present findings are similar to those of Ahmad et al. (2012), who revealed membrane damage in mustard cultivars due to NaCl treatment, which led to modified membrane functioning. However, in comparison among the single/dual inoculation of PGPMs, B3 and B6 reduced the ROS production in Agsun-5264, leading to a significant decrease in membrane lipid peroxidation and enhanced resistibility against stress environment (Han et al., 2014; Lee et al., 2016; Siddiqui et al., 2021). Moreover, the inoculated plants (Agsun-5264) had a higher ROS-detoxifying enzyme activity than non-inoculated plants (Figure 9). Sufficient information indicates that the increased antioxidant enzyme activity plays a significant role in modulating ROS levels in plants during detrimental stress (Gong et al., 2014; Wei et al., 2015; Zhou et al., 2016). Many reports confirm that the capability of microbes and plant cells to reduce the influence of oxidative burst occurs by enhancing the accumulation of antioxidant enzymes and osmolytes (Khan and Bano, 2019; Khanna et al., 2019; Rajput et al., 2019; Vaishnav et al., 2019; Zahir et al., 2019; Nagpal et al., 2020). The findings indicate that the inoculation with B3 and B6 can mitigate the oxidative harm caused by salinity stress. In the present study, cultivar Agsun-5264 inoculated with the isolates B3 and B6 raised SOD and CAT activities during saline stress reduced lipid peroxidation and H_2_O_2_. The result divulges the favorable influence of microbes on the antioxidative enzymes' positive balance, which detoxifies ROS metabolism (Santos et al., 2018).

Higher NiR and NR reductase activity in cultivar Agsun-5264 treated with B3 and B6 supported that the isolated strains are good for crop growth promotion. High nitrogen increases chlorophyll formation and photosynthetic activities (Wu et al., 2021). Likewise, the application of B3 and B6 also raised the nitrite and nitrate content in cultivar Agsun-5264 under salinity stress compared to other treatments. Similar results were observed in leguminous plants while treated with PGPR during abiotic stress (Siddiqui et al., 2021).

The cultivar Agsun-5264 with single/dual inoculation of B3 and B6 demonstrated the highest K+ and the Na+ absorption under a salt-stress environment. It is assumed that the PGPMs stimulated the root system that could increase the plant's ability to utilize higher nutrients, thus rhizosphere pH changes (organic acids), and facilitating K^+^ availability (Lugtenberg et al., 2013; Setiawati and Mutmainnah, 2016). Growth-promoting microorganisms secrete exopolysaccharide compounds, which bind with Na^+^ ions in the root, through which the plant's Na^+^ accretion reduces (Ashraf et al., 2004). Further, PGPMs balance the ion homeostasis and high K^+^/Na^+^ ratios in shoots by decreasing Na^+^ buildup in leaves, rising Na^+^ exclusion through roots, and lifting the high-affinity K^+^ transporter's activity (Ilangumaran and Smith, 2017). Many investigators stated a rise in K^+^ ions uptake in the existence of PGPMs inoculation in alfalfa (Younesi et al., 2013), maize (Rojas-Tapias et al., 2004), and cucumber (Kang et al., 2014). Besides this, Na^+^/K^+^ ratio is a salt tolerance index for plants (Cui et al., 2016). We observed that cultivar Agsun-5264 treated with B3 and B6 had a lower Na^+^/K^+^ ratio under salinity stress conditions, demonstrating that the increased tolerance by the *Bacillus* strain was partially attributable to minimizing Na^+^ accumulation. Our results agreed with the finding of Zhou et al. (2017).

## Conclusion

The current research concludes that the application of bio-inoculants enhances salt tolerance in sunflowers by modulating their water uptake, improving nitrogen assimilation, and better photochemical yield. The smooth electron flow from PSII to photochemical yield, lower photo-inhibition, and reclamation of perturbation in photosynthetic apparatus against salt stress guarded by antioxidant system barricade. Demonstrated essential tolerance mechanisms induced by PGPMs.

## Data Availability Statement

The original contributions presented in the study are included in the article/Supplementary Materials, further inquiries can be directed to the corresponding authors.

## Author Contributions

FA conducted the entire experiment work and prepared illustrations, figures, and write-up. ZSi supervised the research work and guided the manuscript write-up. All authors equally contributed to the research work, manuscript preparation, and illustration.

## Conflict of Interest

The authors declare that the research was conducted in the absence of any commercial or financial relationships that could be construed as a potential conflict of interest.

## Publisher's Note

All claims expressed in this article are solely those of the authors and do not necessarily represent those of their affiliated organizations, or those of the publisher, the editors and the reviewers. Any product that may be evaluated in this article, or claim that may be made by its manufacturer, is not guaranteed or endorsed by the publisher.
