# Supplementary material for: Scrutinizes the Sustainable Role of Halophilic Microbial Strains on Oxygen-Evolving Complex, Specific Energy Fluxes, Energy Flow and Nitrogen Assimilation of Sunflower Cultivars in a Suboptimal Environment
Source: Front Plant Sci. 2022 Jul 18;13:913825. doi: 10.3389/fpls.2022.913825 (PMC9340225; doi:10.3389/fpls.2022.913825)
Supplement: Supplementary file 1 [file Data_Sheet_1.DOCX]

**Table S1.** Different abbreviations, formulations and definitions of selected parameters obtained from JIP protocol for the analysis of chlorophyll fluorescence with their physiological significance presented by (Maxwell and Johnson 2000; Strasser *et al.* 2010, Stirbet *et al.* 2018)

| **Fluorescence**  **Parameters** | **Formula or Equivalence** | | **Physiological Significance** |
| --- | --- | --- | --- |
| ABS/CS |  | Absorption per excitation cross section | |
| ABS/RC | (M0/VJ)/φP0 | Apparent antenna size of active PSII reaction centre | |
| DIo/ABS | 1 – TRo/ABS | Quantum yield of energy dissipation in PSII antenna | |
| DIo/CS | (ABS/CS) – (TR_o_/ CS) | Dissipated energy flux per cross section | |
| DIo/RC | ABS/RC – TR0/RC | Dissipated energy flux per active reaction center | |
| ETo/CS | (ET0/ABS) × (ABS/CS) | The flux of electrons from QA- to PQ per cross section of PSII | |
| ETo/RC | (M0/VJ) × ψE0 | Electron transport per active reaction centre | |
| ETo/TRo | ψo = 1-VJ = (Fm – F2ms)/(Fm – F0) | Efficiency with which a PSII trapped electron is transferred from Quinone A- to Plastoquinone | |
| Fv/Fm | φPo | Maximum quantum yield of primary PSII photochemistry | |
| Fv/Fo | (Fm – F0)/F0 | Size and number of active reactions centre of photosynthetic apparatus | |
| Fo/Fm | φD_0_ | Quantum yield of energy dissipation | |
| PI_ABS_ | RC/ABS ×φPo/(1 – φPo) × ψEo/(1 – ψEo) | Performance index on absorption basis | |
| REo/TRo | = ψR0 = 1 – VI | Efficiency with which a PSII trapped electron is transferred to final PSI  acceptors | |
| TRo/CS | = (TRo/ABS) × (ABS/CS) | Trapped exciton flux per CS | |
| TRo/RC | = Mo/VJ | Maximum trapped exciton flux per active PSII | |
| VJ | (F2ms – F0)/(Fm – F0) | Relative variable Chl a fluorescence at the J-step | |
| VI | (F30ms – F0)/(Fm – F0) | Relative variable Chl a fluorescence at the I-step | |
| Wk | =(Fk−Fo)/(FJ−Fo), | Ratio of variable fluorescence at K-step to the amplitude Fj–Fo | |

**Table S2.** Alterations in percentage of selected parameters of sunflower genotypes with or without PGPMs in response to salt stress environments.

|  | **Agsun-5264** | | | | **S-278** | | | | | |  |  |  |  |  |  |
| --- | --- | --- | --- | --- | --- | --- | --- | --- | --- | --- | --- | --- | --- | --- | --- | --- |
| **%** | **B3** | | **B6** | | **B3** | | **B6** | | | |  |  |  |  |  |  |
|  | **100**  **mM** | **200**  **mM** | **100**  **mM** | **200**  **mM** | **100**  **mM** | **200**  **mM** | **100**  **mM** | | **200**  **mM** | |  |  |  |  |  |  |
| **RWC** | 36 | 40 | 35 | 43 | 19.6 | 19.5 | 15 | | 21 | |  |  |  |  |  |  |
| **S.C** | 258 | 228 | 200 | 231 | 97 | 106 | 126 | | 120 | |  |  |  |  |  |  |
| **CCI** | 54 | 109 | 63 | 95 | 13 | 50 | 20 | | 66 | |  |  |  |  |  |  |
| **O.P** | 69 | 33 | 69 | 29 | 36 | 18 | 30 | | 24 | |  |  |  |  |  |  |
| **F_V_/F_M_** | 11 | 20 | 17 | 25 | 22 | 8 | 22 | | 18 | |  |  |  |  |  |  |
| **PI_ABS_** | 346 | 343 | 292 | 343 | 258 | 96 | 194 | | 185 | |  |  |  |  |  |  |
| **F_V_/F_O_** | 34 | 57 | 61 | 76 | 68 | 15 | 69 | | 46 | |  |  |  |  |  |  |
| **F_O_/F_M_** | 18 | 25 | 26 | 28 | 27 | 9 | 26 | | 24 | |  |  |  |  |  |  |
| **MDA** | 58 | 53 | 60 | 51 | 47 | 34 | 50 | | 45 | |  |  |  |  |  |  |
| **H_2_O_2_** | 53 | 43 | 53 | 42 | 15 | 18 | 17 | | 15 | |  |  |  |  |  |  |
| **CAT** | 114 | 101 | 151 | 110 | 44 | 40 | 76 | | 62 | |  |  |  |  |  |  |
| **SOD** | 120 | 72 | 141 | 74 | 94 | 44 | 141 | | 62 | |  |  |  |  |  |  |
| **Na^+^** | 42 | 43 | 45 | 46 | 29.9 | 29.4 | 31.8 | | 31.6 | |  |  |  |  |  |  |
| **K^+^** | 93 | 102 | 109 | 141 | 47 | 42 | 78.4 | | 78.9 | |  |  |  |  |  |  |
| **Na^+^/K^+^** | 70 | 72 | 73 | 77 | 55 | 51 | 64 | | 62 | |  |  |  |  |  |  |
|  | | | | | |  |  |  | |  |  |  |  |  |  |  |
|  | | | | | |  | |  | | |  |  |  | |  | |


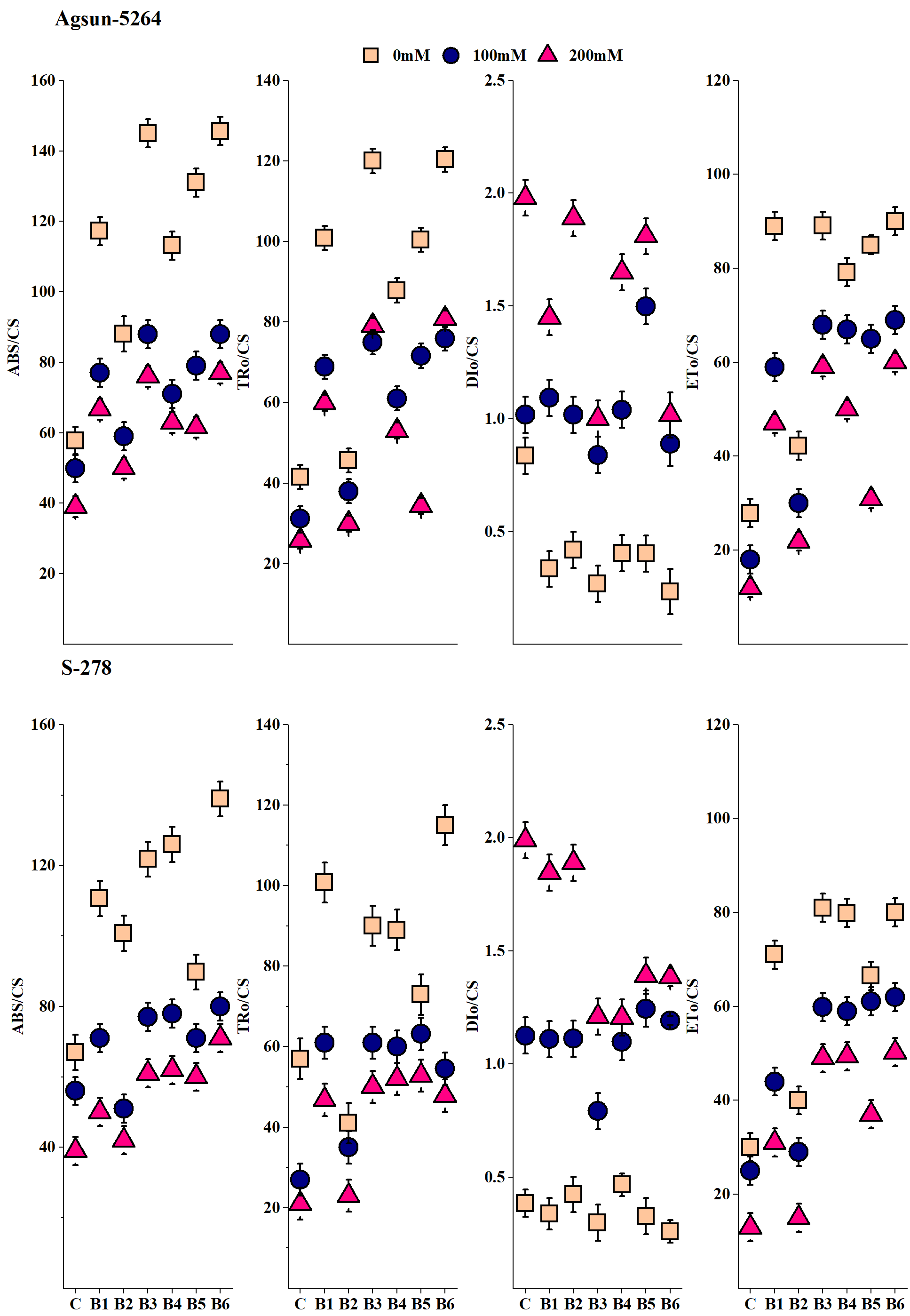
**Fig.S1.** Changes in Phenomenological energy fluxes per CS including ABS/CS, TRo/CS, ETo/CS and DIo/CS of sunflower genotypes under salinity stress after the application of Microbial strains. Vertical drop lines on represented the standard error.

**
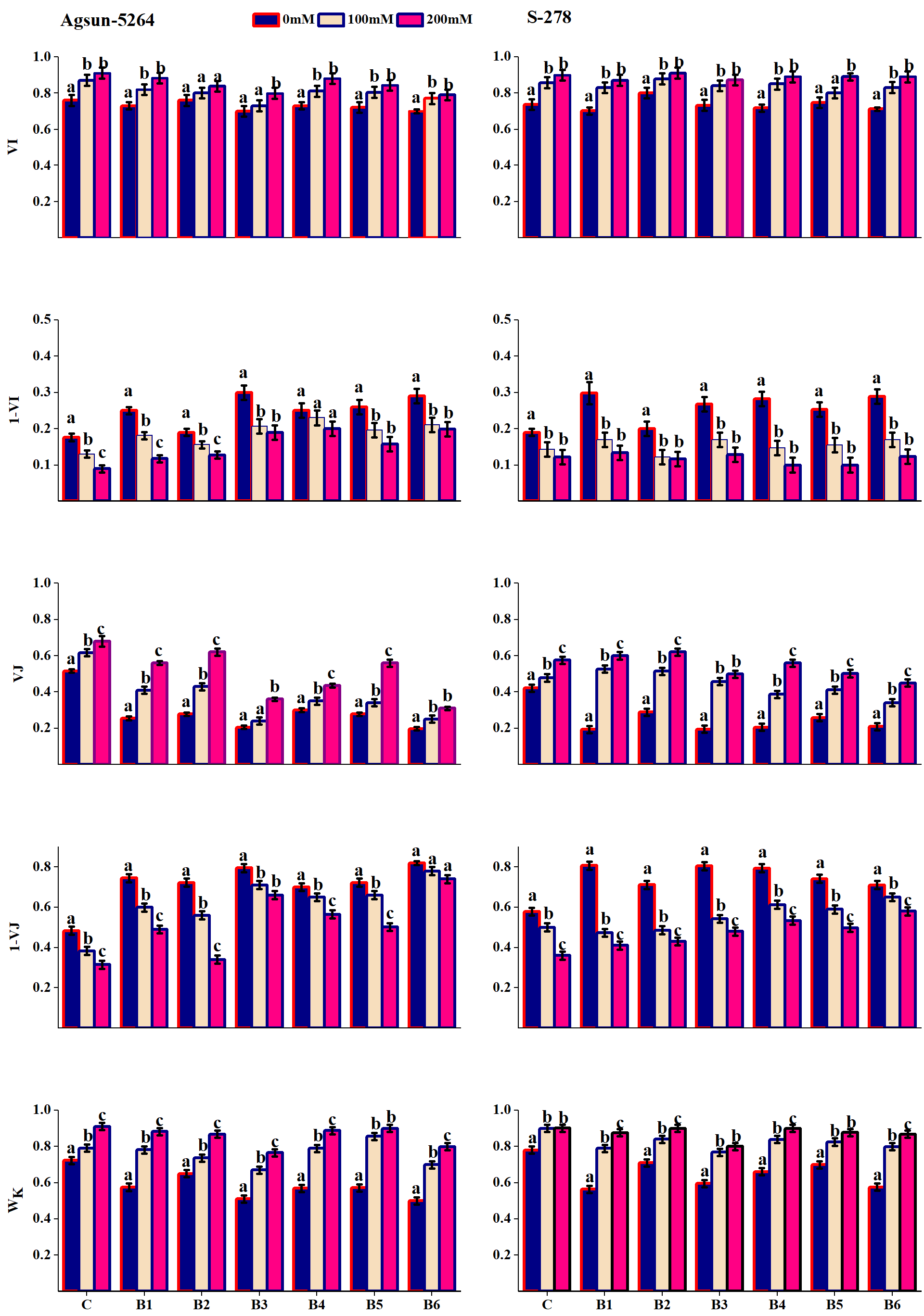
**

**Fig.S2.** Changes in VI, 1-VI, VJ, 1-VJ and W_K_ of sunflower genotypes under salinity stress after the application of Microbial strains. Vertical drop lines on represented the standard error.


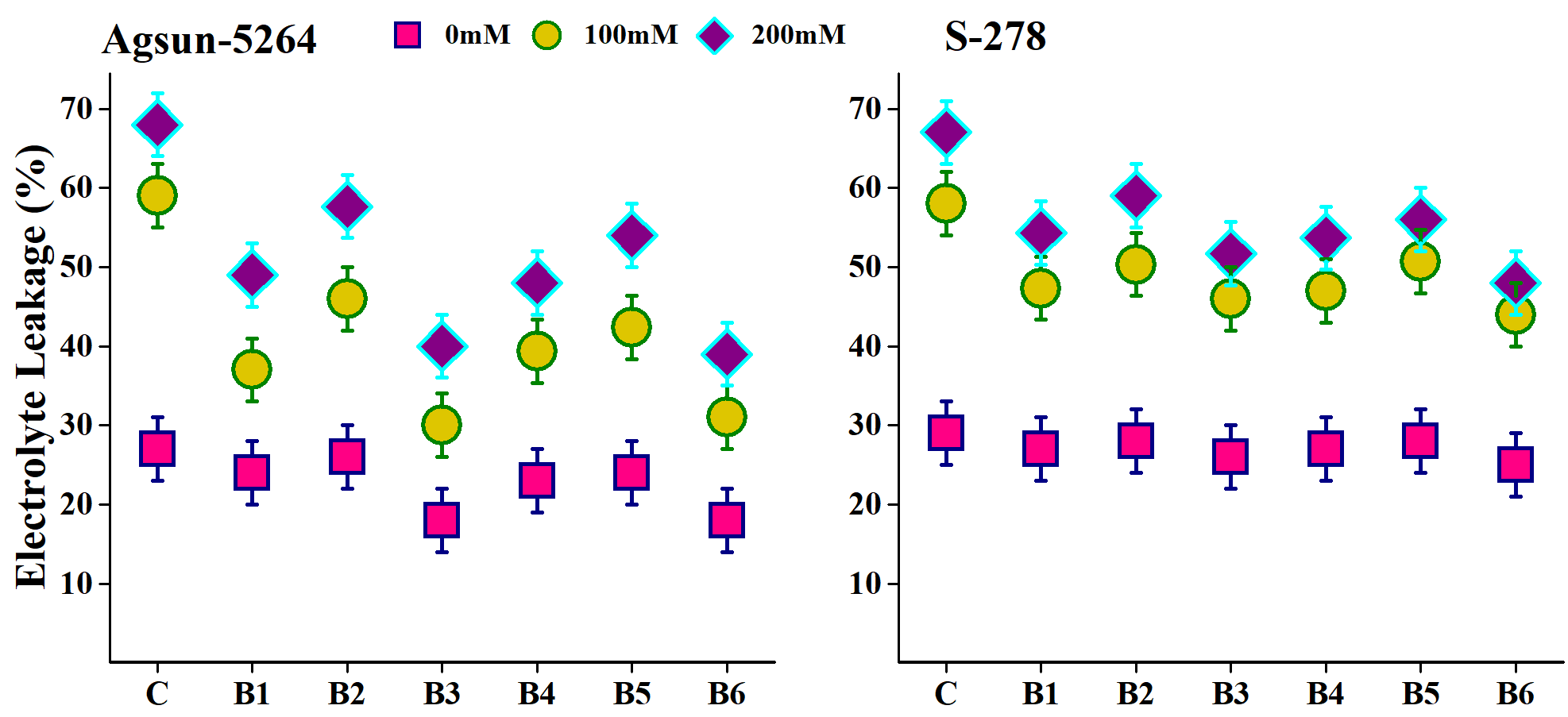
**Fig.S3.** Changes in Electrolyte leakage of sunflower genotypes under salinity stress after the application of Microbial strains. Vertical lines on bar represented the standard error and similar alphabets on error bar expressed the nonsignificant difference at p< 0.05 among the control and salt stress genotypes of sunflower.

**Figure S4a.** Morphological response of sunflower genotypes with or without Microbial strains in response to salt stress environments. **S4b:** Bacillus species cultivation on nutrient agar containing 1M salt strength

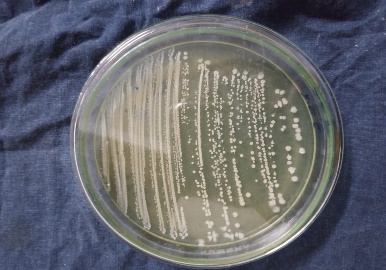

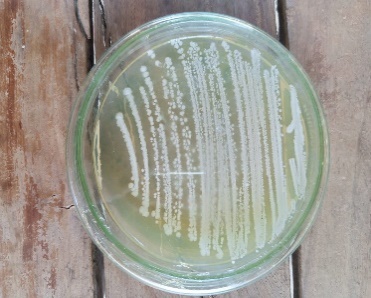

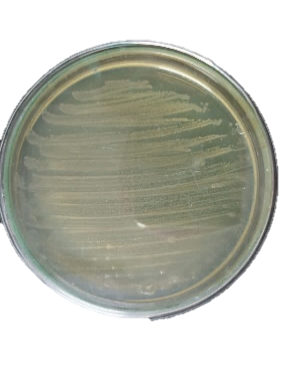

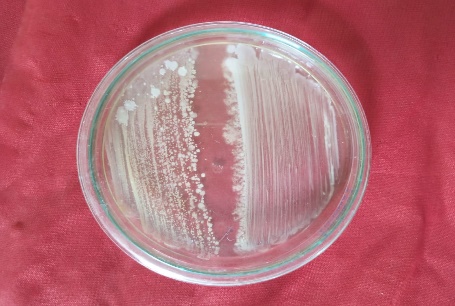

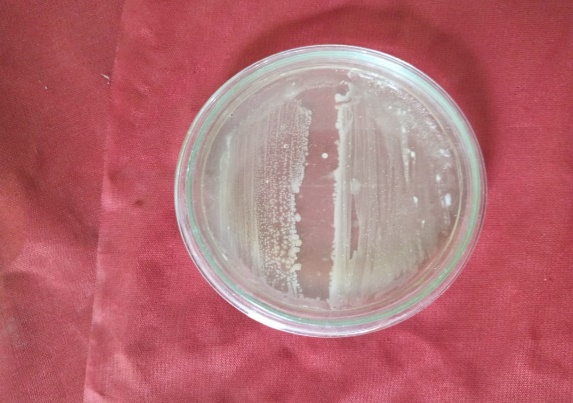

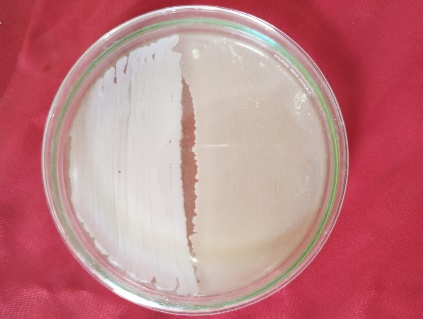


***B.cereus* KUB 27+ *. licheniformis* AAB9**

**B5**

***B.cereus* KUB 15+ *. licheniformis* AAB9**

**B6**

***B.cereus* KUB 15+ KUB 27**

**B4**

***B.cereus* KUB 15**

**B1**

***B.cereus* KUB 27**

**B2**

***B. licheniformis* AAB9**

**B3**
