# Supplementary material for: Scrutinizes the Sustainable Role of Halophilic Microbial Strains on Oxygen-Evolving Complex, Specific Energy Fluxes, Energy Flow and Nitrogen Assimilation of Sunflower Cultivars in a Suboptimal Environment
Source: Front Plant Sci. 2022 Jul 18;13:913825. doi: 10.3389/fpls.2022.913825 (PMC9340225; doi:10.3389/fpls.2022.913825)
Supplement: Supplementary file 2 [file Presentation_1.PPTX]

## Slide 1
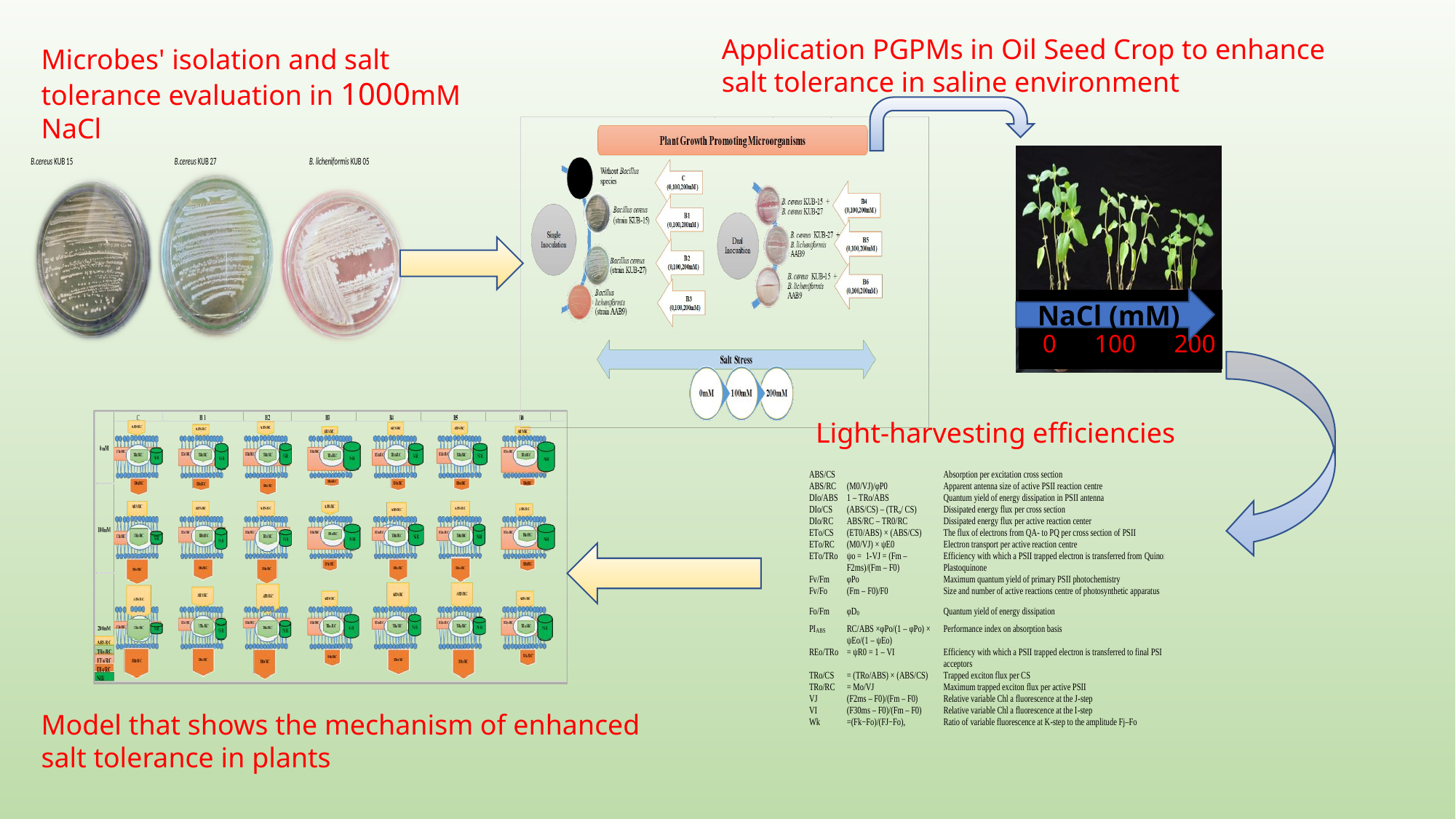

Application PGPMs in Oil Seed Crop to enhance salt tolerance in saline environment
Microbes' isolation and salt tolerance evaluation in 1000mM NaCl
NaCl (mM)
 0 100 200
Light-harvesting efficiencies
Model that shows the mechanism of enhanced
salt tolerance in plants
